# Canal switch in benign paroxysmal positional vertigo: Clinical characteristics and possible mechanisms

**DOI:** 10.3389/fneur.2022.1049828

**Published:** 2022-11-14

**Authors:** Yuexia Wu, Ning Song, Xia Ling, Xiang Li, Yufei Feng, Yue Xing, Ping Gu, Xu Yang

**Affiliations:** ^1^Department of Neurology, Aerospace Center Hospital, Peking University Aerospace School of Clinical Medicine, Beijing, China; ^2^Department of Neurology, The First Hospital of Hebei Medical University, Shijiazhuang, China; ^3^Department of Neurology, Peking University First Hospital, Beijing, China

**Keywords:** benign paroxysmal positional vertigo, canal conversion, canal switch, direction-reversing nystagmus, nystagmus characteristics

## Abstract

**Background:**

Canal switch-benign paroxysmal positional vertigo (CS-BPPV) refers to the phenomenon in which otolith particles move from one canal to another (on the ipsilateral side) during or after canalith repositioning procedure (CRP). However, the clinical characteristics of CS-BPPV and the underlying pathological mechanisms remain unclear. In this study, we investigated the incidence of canal switch (CS) for the different semicircular canals in benign paroxysmal positional vertigo (BPPV), examined nystagmus characteristics, and explored the underlying mechanisms.

**Methods:**

Clinical data for 1,099 patients with single-canal BPPV were collected and retrospectively analyzed. The incidences of CS in the different types of BPPV were analyzed. Patients were divided into CS-BPPV and non-CS (NCS)-BPPV groups according to whether they exhibited CS after CRP. The baseline characteristics and nystagmus characteristics of patients were compared between the two groups.

**Results:**

Patients with BPPV who developed or did not develop CS accounted for 4.6% (51/1,099) and 95.4% (1,048/1,099), respectively, of the patients included in the study. There were no statistically significant differences between the two groups in terms of sex, age, side of the canals involved, hypertension, or diabetes. CS was observed in 3.7% (25/677) of patients with PC-BPPV, including conversion between posterior canal (PC) and horizontal canal (HC) (1.6%, 11/677), and between PC and anterior canal (AC) (2.1%, 14/677). CS was observed in 5.2% (17/327) of patients with HC-BPPV, including from HC to PC (4.3%, 14/327), and from HC to AC (0.9%, 3/327). CS was found in 9.5% (9/95) of patients with AC-BPPV, including from AC to PC (8.4%, 8/95), and from AC to HC (1.1%, 1/95). The intensity of nystagmus was significantly greater in the CS-BPPV group compared with that in the NCS-BPPV group [24.00 (11–39) vs. 12.00 (7–24), *P* < 0.001]. Furthermore, the incidence of direction-reversing nystagmus was significantly higher in the CS-BPPV group than in the NCS-BPPV group [31.4% (16/51) vs. 4.3% (45/1,048), *P* < 0.001].

**Conclusions:**

CS in BPPV is uncommon. Patients with AC-BPPV are more likely to develop CS, followed by patients with HC-BPPV and PC-BPPV. The occurrence of CS-BPPV may be related to the anatomical structure of the semicircular canals. When the canals contain large/heavy accumulations of otolith particles, CS may be more common during re-examination after CRP.

## Introduction

Benign paroxysmal positional vertigo (BPPV) is a paroxysmal, transient episode of vertigo triggered by specific head position changes. BPPV is one of the most common causes of dizziness/vertigo, accounting for approximately 24.1% of all cases (1). The lifetime prevalence of BPPV is 2.4%, the 1-year prevalence is 1.6%, and the 1-year incidence is 0.6% (2). In BPPV, the otoliths dislodge from the utricle because of utricular degeneration or head trauma, or other reasons (3). The otolith fragments float freely in the semicircular canals (canalolithiasis) or adhere to the cupula of a semicircular canal (cupulolithiasis) (4). One or more of the three semicircular canals can be involved. Clinically, according to the semicircular canals involved, canalith repositioning procedure (CRP) can be performed to move the detached otoliths from the canal to the utricle. Currently, CRP is the standard treatment for BPPV, with apparent therapeutic efficacy (5, 6). Approximately 80% of BPPV patients experience symptom relief after a single CRP session (7). However, during or after CRP, otolith particles can move from one canal to another on the ipsilateral side, without repositioning to the utricle, resulting in canal switch (CS)-BPPV. If CS-BPPV is not recognized, it can prolong the duration of BPPV and increase the risk of falls in patients. Foster et al. (8) performed CRP in 44 patients with posterior canal (PC)-BPPV, and found that performing the Dix–Hallpike test immediately after CRP increased the risk of CS. A study conducted by Park et al. (9) on 709 patients with anterior canal (AC)- or PC-BPPV showed that transition from AC- to PC-BPPV occurred; however, the factors causing CS were unclear. Lee et al. (10) investigated the occurrence of CS in patients with PC-BPPV and horizontal canal (HC)-BPPV, and found that CS was significantly associated with the use of multiple CRP sessions and the Gufoni maneuver. However, studies of CS-BPPV are limited and non-systematic, and therefore, the occurrence of CS between HC and PC, and between PC and AC, in addition to the mechanisms underlying BPPV, remain unclear.

In this study, we investigate the incidence of CS in the different types of BPPV. Furthermore, we examine the link between CS-BPPV and nystagmus characteristics, and we explore the underlying mechanisms. Our findings should aid clinicians in the diagnosis and treatment of BPPV.

## Materials and methods

### Subjects

We retrospective recruited 1,099 patients with a first episode of single-canal BPPV within 6 months who visited the Vertigo Clinic of our hospital between April, 2017 and November, 2021. Patients diagnosed with single-canal BPPV (including canalolithiasis and cupulolithiasis) according to the diagnostic criteria established by the International Bárány Society, 2015 (11), were included in the study. Patients with multiple-canal BPPV, vestibular migraine, positional vertigo or nystagmus caused by central lesions (such as lesions involving the brainstem, cerebellum and the vicinity of the fourth ventricle), and patients unable to tolerate the position tests or CRP were excluded.

Eye movement tests, position tests (supine roll test, Dix-Hallpike test, straight head hanging test) were performed in all patients by a single senior technician. Nystagmus was recorded using a videonystagmography system (Interacoustics, Assens, Denmark). During the position tests, patients were maintained in each position for more than 1 min to record the changes in nystagmus, and to observe whether direction-reversing nystagmus occurred.

The studies involving human participants were reviewed and approved by the Ethics Committee of Aerospace Center Hospital, Peking University Aerospace School of Clinical Medicine. The patients provided their written informed consent to participate in this study.

### Manual CRPs and the diagnosis of CS-BPPV

According to the semicircular canals involved, manual CRPs (Barbecue maneuver for HC-BPPV, Epley maneuver for PC-BPPV, and Yacovino maneuver for AC-BPPV) were performed by a single senior physician. The positional tests were re-examination 30 min or the next day after CRP.

CS was diagnosed when transition from HC-BPPV to PC-BPPV, PC-BPPV to HC-BPPV, PC-BPPV to AC-BPPV, AC-BPPV to PC-BPPV, HC-BPPV to AC-BPPV, or AC-BPPV to HC-BPPV was observed.

The incidence of CS in patients with different types of BPPV was also examined. According to whether CS occurred after CRP, patients were divided into CS-BPPV and non-CS (NCS)-BPPV groups, and sex, age, side of the semicircular canal involved, hypertension, diabetes, nystagmus intensity and the incidence of direction-reversing nystagmus were compared between the two groups.

### Statistical analysis

All statistical analyses were performed with SPSS 20.0 software. Continuous variables were expressed as mean ±standard deviation (SD), comparisons for normally distributed data between groups were performed by using independent sample *t*-test. The categorical variables were expressed as percentages, and comparisons between groups were performed using the chi-square (χ2) test with Yates' continuity correction or Fisher's exact test, as appropriate. All reported *P* values are two-tailed, *P* < 0.05 was considered statistically significant.

## Results

### Clinical characteristics of patients included in the study

Among the 1,099 patients with BPPV included in the study, CS was observed in 51 patients, including 38 females with an average age of 60.00 ± 14.00 years. CS was not observed in 1,048 patients, including 739 females with an average age of 59.00 ± 21.00 years. In the CS-BPPV group, 18 patients had left-sided BPPV, and 33 patients had right-sided BPPV. In the NCS-BPPV group, 434 patients had left-sided BPPV, and 614 patients had right-sided BPPV. There were no statistically significant differences between the two groups in sex, age side of the canals involved, hypertension, or diabetes (Table 1).

**Table 1:** Clinical baseline characteristics of patients with CS-BPPV and NCS-BPPV.

	**CS-BPPV (*n* = 51)**	**NCS-BPPV (*n* = 1,048)**	***p*-value**
Sex, *n* (%)			*P* = 0.540
Male	13 (25.5%)	309 (29.5%)	
Female	38 (74.5%)	739 (70.5%)	
Age, mean ± SD, years	60.00 ± 14.00	59.00 ± 21.00	*P* = 0.159
Canal side, *n* (%)			*P* = 0.386
Left	18 (35.3%)	434 (41.4%)	
Right	33 (64.7%)	614 (58.6%)	
Hypertension (yes)	13 (25.5%)	262 (25.0%)	*P* = 0.937
Diabetes (yes)	5 (9.8%)	131 (12.5%)	*P* = 0.568

CS, canal switch; NCS, Non-canal switch.

### Incidence and type of CS-BPPV

**Figure 1:**
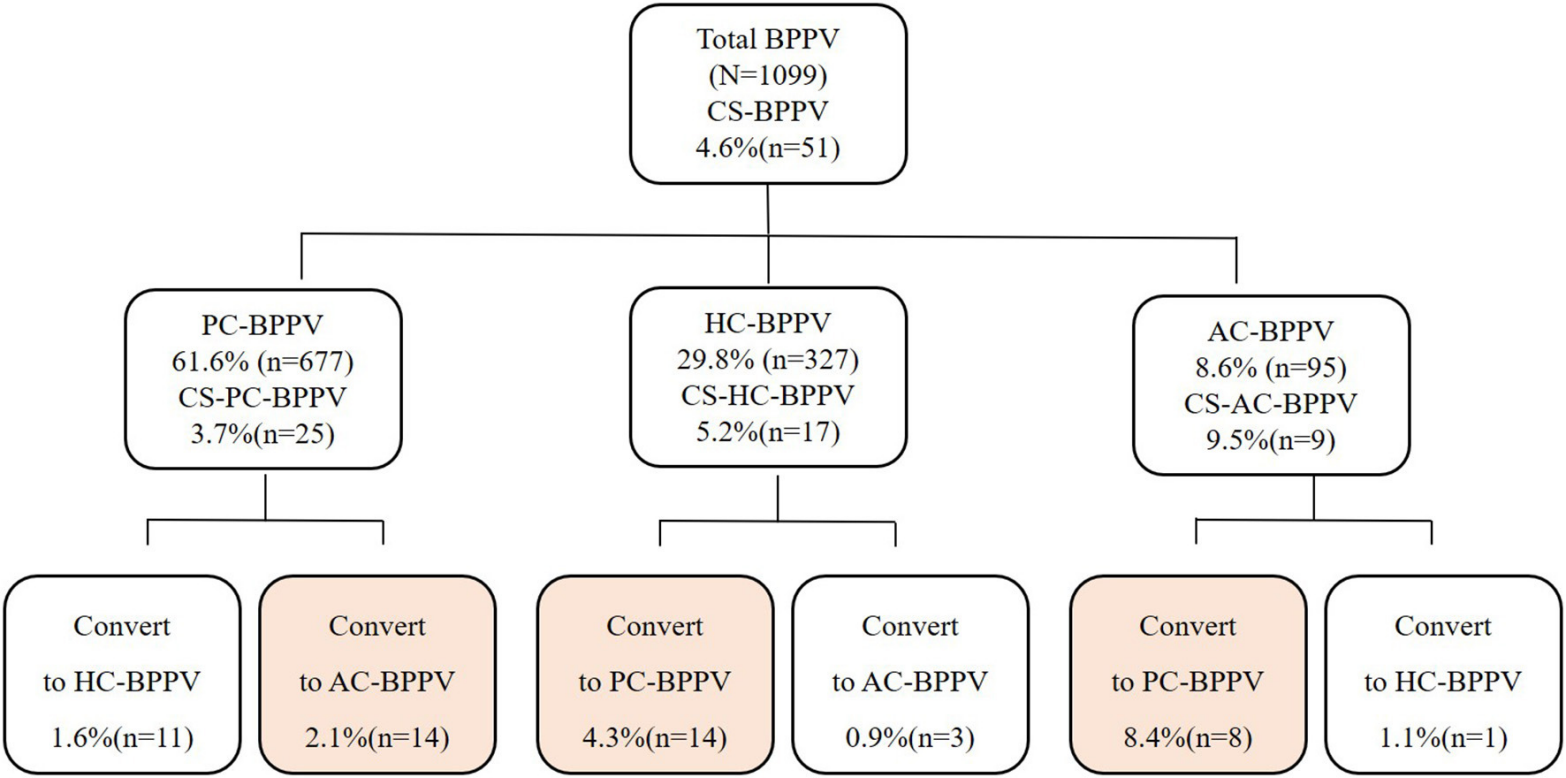
The distribution of canal switch in BPPV. BPPV, benign paroxysmal positional vertigo; CS, canal switch; PC, posterior canal; HC, horizontal canal; AC, anterior canal.

BPPV patients with and without CS accounted, respectively, for 4.6% (51/1,099) and 95.4% (1,048/1,099) of all patients. Among these, 677 had PC-BPPV, accounting for 61.6% (677/1,099) of all patients included, and CS was observed in 3.7% (25/677) of these patients with PC-BPPV, including from PC to HC in 1.6% (11/677) of patients, and from PC to AC in 2.1% (14/677) of patients. Among the patients, 327 had HC-BPPV, accounting for 29.8% (327/1,099) of all patients, and CS was observed in 5.2% (17/327) of patients with HC-BPPV, including from HC to PC in 4.3% (14/327) of patients, and from HC to AC in 0.9% (3/327) of patients. Among the participants, 95 had AC-BPPV, accounting for 8.6% (95/1,099) of all patients included, and CS was found in 9.5% (9/95) of patients with AC-BPPV, including from AC to PC in 8.4% (8/95) of patients, and from AC to HC in 1.1% (1/95) of patients (Figure 1). Furthermore, CS was found in 25, 17 and 9 patients with PC-, AC- and HC-BPPV, respectively, accounting for 2.3% (25/1,099), 1.5% (17/1,099) and 0.8% (9/1,099), respectively, of all patients included in the study. CS from PC to HC, PC to AC, HC to PC, HC to AC, AC to PC, and AC to HC accounted, respectively, for 1.0% (11/1,099), 1.3% (14/1,099), 1.3% (14/1,099), 0.3% (3/1,099), 0.7% (8/1,099) and 0.1% (1/1,099) of all cases.

### Nystagmus characteristics

**Figure 2:**
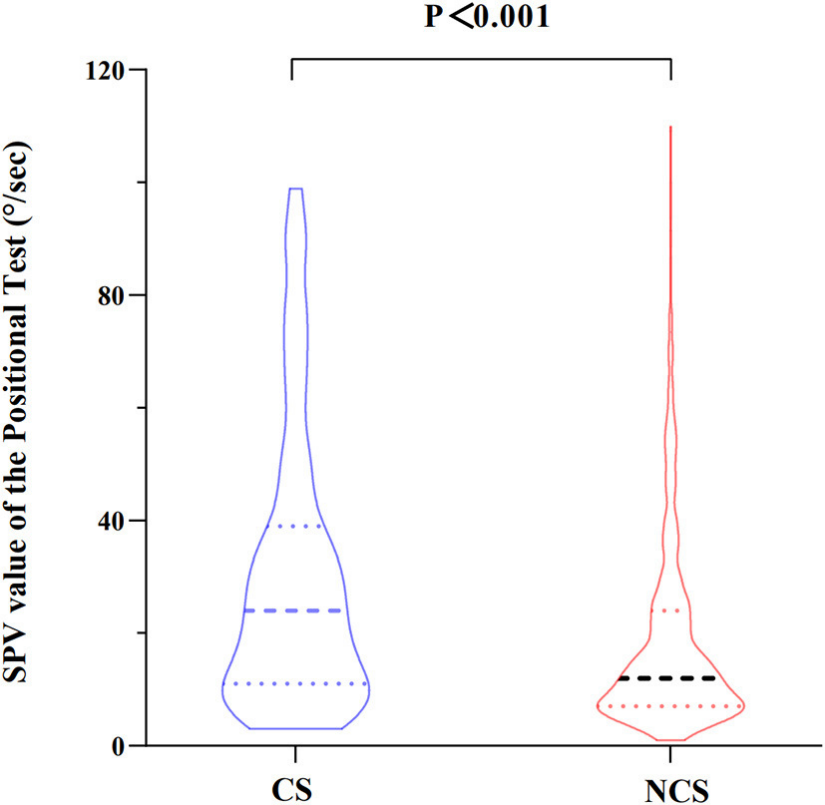
Comparison of maximum slow phase velocity (SPV) of the nystagmus evoked by the position tests between the two groups.

The maximum slow phase velocity (SPV) of the nystagmus evoked by the position tests in patients with different types of BPPV was compared. The nystagmus intensity (maximum SPV) evoked by the position tests was significantly greater in the CS-BPPV group than that in the NCS-BPPV group [24.00 (11–39) vs. 12.00 (7–24), *P* < 0.001] (Figure 2).

Direction-reversing nystagmus was noted in 31.4% (16/51) of patients in the CS-BPPV group and 4.3% (45/1,048) of patients in the NCS-BPPV group, and a statistically significant difference was found between the two groups (*P* < 0.001).

## Discussion

In this study, while CS was uncommon in patients with BPPV, patients with AC-BPPV had a high probability of CS, followed by patients with HC-BPPV and PC-BPPV. The incidence of direction-reversing nystagmus was higher in CS-BPPV patients than in NCS-BPPV patients, when they underwent position tests.

### CS from AC to PC

**Figure 3:**
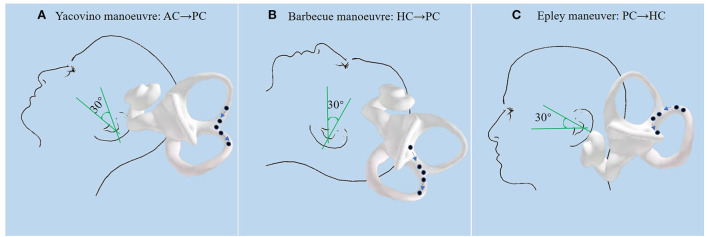
**(A)**: During the Yacovino maneuver, when step 3 (flexing the patient's neck to the chin-to-chest position) is completed and the next step is not performed in a timely manner, the otolith particles enter the PC through the common crus. **(B)**: During the Barbecue maneuver for HC-BPPV, if the patient is not brought from the supine position to the sitting position, the otolith particles that have been repositioned into the utricle enter the PC through the common crus under the action of gravity, which then enter the ampullary end of the PC when sitting up. **(C)**: During the last step of the Epley maneuver (sitting up), otolith particles enter the HC because of tilting of the head to the affected side after sitting up.

In this study, CS from AC-BPPV to PC-BPPV was observed in 8.4% (8/95) of all patients with AC-BPPV, the most common type of CS-BPPV. CS from PC-BPPV to AC-BPPV was observed in 2.1% (14/677) of all patients with PC-BPPV. In a previous study (9), CS from AC-BPPV to PC-BPPV accounted for 12.1% of all AC-BPPV patients, and CS from PC-BPPV to AC-BPPV accounted for 2.3% of all PC-BPPV patients. Our present results are in agreement with these previous reports. Although the proportion of patients with AC-BPPV is much lower than those with PC-BPPV, the probability of CS was much higher in AC-BPPV than in PC-BPPV. This may be related to the unique anatomical structure of the AC; i.e., the ends of the AC and PC join to form the common crus at the non-ampullary end. In this study, patients with AC-BPPV were treated with the Yacovino maneuver, which consists of the following four steps (12): step 1, sit straight; step 2, bring the head to a hanging position, 30° below the horizontal plane; step 3, flexing the neck to the chin-to-chest position; and step 4, returning to the sitting position. We speculate that CS from AC-BPPV to PC-BPPV may occur when step 3 of the Yacovino maneuver is kept for a longer duration, resulting in otolith particles entering the PC through the common crus (Figure 3A). Findings from a recent study (13) support this concept. Therefore, to avoid CS, we recommend that the patient's head be elevated 45° above the horizontal plane during step 3 of the Yacovino maneuver, or alternatively, that a modified Yacovino maneuver, proposed by Bhandari et al. (13), is applied; i.e., the patient is brought directly from the step 2 to the sitting position. After an interval of 30 s, the neck of the patient is flexed forward at an angle of 45°.

Typically, the Epley maneuver consists of five steps (14): step 1, the patient is positioned in the sitting position with the head turned 45° to the affected side; step 2, the patient is rapidly laid down with the head hanging 15–30° below the bed; step 3, the head is rotated 90° to the healthy side; step 4, the body is turned 90° to the healthy side, with the head turned 135° to the healthy side, with the nose facing downwards; step 5, the patient is returned to the sitting position. Because the ends of the AC and PC join at the non-ampullary end to form the common crus, we speculate that the otolith particles may translocate from the joining point of the common crus to the AC during step 4 of the Epley maneuver. The findings from the study of Yong et al. (15) support this concept. Yong et al. showed that in three patients with CS from PC-BPPV to AC-BPPV, vertical up-beating nystagmus was observed during step 4 of Epley maneuver. The up-beating nystagmus was likely induced by otolith particles entering the AC and migrating toward the ampulla. Therefore, we recommend that the Epley maneuver be performed accurately. Tilting of the patient's head downwards excessively under the action of gravity should be avoided during step 4, to reduce the translocation of otolith particles into the AC.

### CS between HC and PC

In this study, CS from HC-BPPV to PC-BPPV accounted for 4.3% (14/327) of patients with HC-BPPV, and CS from PC-BPPV to HC-BPPV accounted for 1.6% (11/677) of patients with PC-BPPV. A study conducted by Lee et al. (10) showed that the incidence of CS from HC-BPPV to PC-BPPV and CS from PC-BPPV to HC-BPPV was 3.5 and 2.4%, respectively. In a study investigating the efficacy of CRP in 58 patients with PC-BPPV, rapid transition of PC-BPPV to HC-BPPV was found in two patients (16). In a prospective study of 72 HC-BPPV patients treated with modified 360° CRP, four patients developed CS, and symptoms and nystagmus subsided following CRP for PC-BPPV (17).

Our findings show that the incidence of CS from HC-BPPV to PC-BPPV was higher than that of CS from PC-BPPV to HC-BPPV. The reason may be that during the Barbecue maneuver, few doctors use a 30° pillow, and therefore, patients are in the supine position after the 360° rotation, allowing otolith particles that have been repositioned into the utricle to enter the PC through the common crus under gravity (Figure 3B). A study (18) suggested that CS is more likely to occur during CRP when the head is not kept 30° above the horizontal plane. A prospective study of patients with HC-BPPV treated with the Gufoni maneuver (19) showed that the incidence of CS from HC-BPPC to PC-BPPV was as high as 13.8%.

In this study, the incidence of CS from PC-BPPV to HC-BPPV was 1.6%. Yong et al. (15) reported that PC-BPPV can be converted to HC-BPPV during steps 3 and 4 of the Epley maneuver; however, the probability is very small because of the anatomical position of the canal. We speculate that the otolith particles may enter the HC because of the tilting of the head to the affected side after sitting up during step 5 of the Epley maneuver (Figure 3C). Therefore, we recommend that for patients with PC-BPPV, the head should be tilted forward and toward the healthy side during the last step of the Epley maneuver. A randomized controlled study (20) showed that increasing the number of accelerations and decreasing the rotation angle can reduce the occurrence of CS in PC-BPPV during the Epley maneuver.

A study (21) showed that among 138 pateints with PC-BPPV treated by computer-controlled CRP, CS from PC-BPPV to HC-BPPV occurred in 1.5% of the patients. Although the incidence of CS from PC-BPPV to HC-BPPV in the present study is similar, CS following manual CRP may not reflect the conditions of computer-controlled CRP. Therefore, further studies are needed to clarify the incidence of CS in patients with BPPV treated by machine CRP.

### CS between the HC and AC

CS between the HC and AC is less common. In the present study, CS from HC-BPPV to AC-BPPV accounted for 0.9% (3/327) of patients with HC-BPPV, and CS from AC-BPPV to HC-BPPV accounted for 1.1% (1/95) of patients with AC-BPPV. Because of the extremely low incidence, CS between the HC and AC has rarely been studied. Our findings demonstrate the occurrence of CS between HC-BPPV and AC-BPPV; however the underlying mechanism is unclear. We speculate that CS between HC-BPPV and AC-BPPV may occur during the Barbecue maneuver, and that the otolith particles may enter the AC when the patient is in the prone position (lying on the healthy side). CS between HC-BPPV and AC-BPPV may also occur after CRP, i.e., after the otolith particles have been repositioned to the utricle, they may fall into the AC because of head movements (e.g. head bowing).

### CS in BPPV may be associated with large/heavy accumulations of otolith particles

In the present study, we found that the intensity of the nystagmus evoked by the position tests was significantly greater in the CS-BPPV group than in the NCS-BPPV group (Supplementary Video 1). Additionally, the incidence of direction-reversing nystagmus was also significantly higher in the CS-BPPV group compared with that in the NCS-BPPV group. A previous study showed that direction-reversing nystagmus during position tests may be caused by short-term central adaptation following the intense first phase nystagmus (22). This suggests that the high SPV observed in the CS-BPPV group is not accidental.

Studies have shown that the SPV of nystagmus evoked by the position tests is not only related to the skills of the technicians, the angle between the semicircular canal plane and the gravity vector during the position movement, and the distance that the otolith fragments move (23, 24), but also to the number, size and density of the otolith particles (25). Larger or heavier otolith particles may lead to higher SPV of nystagmus evoked by the position tests. A mathematical model of BPPV (26) showed that large numbers of small otolith particles are more likely to cause intense nystagmus than small numbers of large otolith particles, indicating that large numbers of small otolith particles can also lead to a high SPV of nystagmus during position tests. In this study, position tests were performed by a fixed senior technician, and therefore, the impact of the technician's skills on the recorded SPV is likely negligible. Considering the influence of otolith particles, the following two factors may cause intense nystagmus during the position test: the accumulation of otolith particles (larger volume or mass) and dispersion of otolith particles (large number of dispersed small otolith particles). Foster et al. (8) showed that patients effectively treated with a single cycle of CRP may have larger or greater accumulation of otolith particles, and that these patients may have high probability of CS and canal re-entry. Clinically, BPPV caused by accumulation of otolith particles is mostly single-canal BPPV, and CRP is effective in these patients, while BPPV caused by dispersion of otolith particles is mostly multi-tube BPPV, and multiple CRPs are often required. Therefore, we speculate that when large/heavy accumulation of otolith particles is present, patients may be more likely to develop CS during the re-examination of nystagmus after CRP.

### Limitations

This study has some limitations. First, this is a retrospective study, and the occurrence of CS in BPPV during CRP was not observed in a systematic manner. Second, the impact of the number of BPPV episodes and the total duration of disease on the occurrence of CS was not investigated, and further studies are needed to clarify their influence. Third, in-depth analysis of the risk factors associated with CS-BPPV was not performed because of the lack of etiological data.

## Conclusions

Our findings suggest that while CS is uncommon in patients with BPPV, patients with AC-BPPV had a high probability of CS, followed by patients with HC-BPPV and PC-BPPV. The occurrence of CS-BPPV may be related to the anatomical structure of the semicircular canals. When large/heavy accumulations of otolith particles are present, CS may be more likely to occur during re-examination after CRP.

## Data availability statement

The original contributions presented in the study are included in the article/Supplementary material, further inquiries can be directed to the corresponding authors.

## Ethics statement

The studies involving human participants were reviewed and approved by the Ethics Committee of Aerospace Center Hospital, Peking University Aerospace School of Clinical Medicine. The patients/participants provided their written informed consent to participate in this study.

## Author contributions

XY contributed to the conception and design of the study. XLin, XLi, YF, and YX collected the clinical data. YW and NS analyzed the results, drafted, and corrected the manuscript. All authors contributed to the article and approved the submitted version.

## Funding

This study was supported by Aerospace Center Hospital (HP2021-03-50703).

## Conflict of interest

The authors declare that the research was conducted in the absence of any commercial or financial relationships that could be construed as a potential conflict of interest.

## Publisher's note

All claims expressed in this article are solely those of the authors and do not necessarily represent those of their affiliated organizations, or those of the publisher, the editors and the reviewers. Any product that may be evaluated in this article, or claim that may be made by its manufacturer, is not guaranteed or endorsed by the publisher.

## Supplementary material

Supplementary video 1 A patient with HC-BPPV with intense nystagmus showing canal switch to the PC after CRPs.
